# Integrated beamline control and data acquisition for small-angle X-ray scattering at the P12 BioSAXS beamline at PETRAIII storage ring DESY

**DOI:** 10.1107/S1600577518005398

**Published:** 2018-04-25

**Authors:** Nelly R. Hajizadeh, Daniel Franke, Dmitri I. Svergun

**Affiliations:** a Hamburg Outstation, European Molecular Biology Laboratory, Notkestrasse 85, 22607 Hamburg, Germany

**Keywords:** SAXS, beamline, control system, data collection, *BECQUEREL*, alignment

## Abstract

The versatility of small-angle X-ray scattering (SAXS) as a structural biology method is apparent by its compatibility with many experimental set-ups. Most advanced SAXS studies are conducted at dedicated synchrotron beamlines yielding high beam brilliance, throughput and temporal resolution. However, utilizing the full potential of the method while preserving a high degree of automation provides a challenge to any SAXS beamline. This challenge is especially pertinent at the P12 BioSAXS beamline of the EMBL at the PETRAIII Synchrotron DESY (Hamburg, Germany), optimized and dedicated to scattering of macromolecular solutions. Over 200 unique set-ups are possible at this beamline offering various functionalities, including different temporal and spatial resolutions. Presented here is a beamline control and data-acquisition software, *BECQUEREL*, designed to maximize flexibility and automation in the operation of P12. In the frame of a single intuitive interface the control system allows for convenient operation with all hardware set-ups available at P12 including a robotic sample changer, in-line size-exclusion chromatography, stop-flow devices, microfluidic spinning disk and various in-air settings. Additional functionalities are available to assist the data-collection procedure for novice users, and also routine operation of the support staff.

## Abstract 2

A versatile C++/Python-based data-acquisition and beamline control system for the P12 small-angle X-ray scattering beamline P12 at PETRAIII (DESY, Hamburg, Germany) is presented.

## 1. Introduction   

Since the first biological small-angle X-ray scattering (SAXS) experiments were conducted in the 1950s (Guinier & Fournet, 1955 ▸), the technique has developed to the point where most synchrotron sources have dedicated SAXS beamlines, and many of those are partly or fully dedicated to biological experiments (Blanchet *et al.*, 2015 ▸; Pernot *et al.*, 2013 ▸; Li *et al.*, 2016 ▸; Acerbo *et al.*, 2015 ▸; Labrador *et al.*, 2013 ▸; David & Pérez, 2009 ▸). The popularity of SAXS for structural biology can be ascribed to its ability to extract information from macromolecules, under a variety of conditions and in a high-throughput manner (Graewert & Svergun, 2013 ▸; Skou *et al.*, 2014 ▸). The widespread presence of SAXS beamlines at major light sources can also be attributed to advances in the experimental procedure, especially in terms of automation of hardware as well as to the process of data reduction. Examples of such include robotic sample handling, data-analysis pipelines, and modelling software (Franke *et al.*, 2012 ▸, 2017 ▸; Hura *et al.*, 2009 ▸; Brennich *et al.*, 2016 ▸; Hopkins *et al.*, 2017 ▸). These developments, coupled with improvements in source brightness and low-noise detectors (Broennimann *et al.*, 2006 ▸; Johnson *et al.*, 2014 ▸), have extended the scope of experimental set-ups, making it possible to answer a much broader set of biological questions. The sample can be delivered in various ways, using, for example, high-throughput pipetting robots (Round *et al.*, 2015 ▸), size-exclusion or ion-exchange chromatography columns (SEC and IOC, respectively) (David & Pérez, 2009 ▸; Hutin *et al.*, 2016 ▸; Graewert *et al.*, 2015 ▸), microfluidic chips (Schwemmer *et al.*, 2016 ▸; Jain *et al.*, 2013 ▸) or stop-and-flow devices for rapid mixing and kinetic studies (Angelov *et al.*, 2015 ▸; Blanchet *et al.*, 2015 ▸; Kathuria *et al.*, 2011 ▸).

At the P12 BioSAXS beamline at the PETRAIII storage ring (DESY, Hamburg, Germany) a diverse range of experimental set-ups are available (Blanchet *et al.*, 2015 ▸). The most common modes feature a high-throughput robotic sample changer (Round *et al.*, 2015 ▸) and SEC-SAXS with additional biophysical characterization [refractive index, right-angle light scattering, ultraviolet (RI/RALLS/UV)] (Graewert *et al.*, 2015 ▸). More infrequent set-ups include a stopped-flow device for fast time-kinetics, microfluidic SAXS disk (Schwemmer *et al.*, 2016 ▸) for ultra-small-volume handling as well as multiple in-air set-ups and an anomalous SAXS (ASAXS) mode. For all set-ups, P12 can be utilized with four types of Dectris detectors, Pilatus 1MW, Pilatus 2M, Pilatus 6M (Broennimann *et al.*, 2006 ▸) and Eiger 4M (Johnson *et al.*, 2014 ▸), covering different angular ranges and offering different temporal resolution. Moreover, two types of monochromators can be used, a double-crystal Si(111) monochromator or a double-multilayer providing about 50 times higher flux (5 × 10^14^ photons s^−1^). Additional flexibility is introduced by the adjustable detector-tube, allowing a total of five distances and therefore different angular spacings and ranges. Taken together, the combination of possible set-ups at P12 amounts to more than 200 settings offering various functionalities, background levels, temporal and spatial resolution.

Accommodating such a diverse range of experimental set-ups and procedures with their associated requirements on hardware synchronization and data collection presents a significant challenge. This challenge becomes especially relevant in the context of optimizing the available beam time both in terms of sample throughput and data quality as well as the number of user access hours. Different experiments may require their own sample environments and/or adjustments of the upstream beamline components. If the experiment also entails an exchange of a monochromator and/or a detector, yet more components are affected. To allow for such flexibility without compromising robustness and beam quality, the need for a comprehensive beamline control and data-acquisition software becomes apparent.

At the P12 BioSAXS beamline, the graphical user interface (GUI) of *BECQUEREL* (*Biosaxs Experiment Control QUEue and RELax*) provides the necessary combination of flexibility and automation to efficiently operate a multi-set-up beamline. All devices at the beamline, from the storage ring to the detector, are available to the user through this GUI. This same GUI is also used to set up and trigger fully automated data collections. Combining beamline control and data acquisition in one interface brings more advantages than simply a unified interface for all tasks. It also means that all devices are by default able to be included in the data-collection procedure, opening up the possibility to automate procedures with beamline optical elements during a data collection. Here, we describe the advantages of such a comprehensive system, and in particular how it aids the operation of a multi-set-up BioSAXS beamline for the users as well as for the support staff.

## 2. Software architecture   

**Figure 1:**
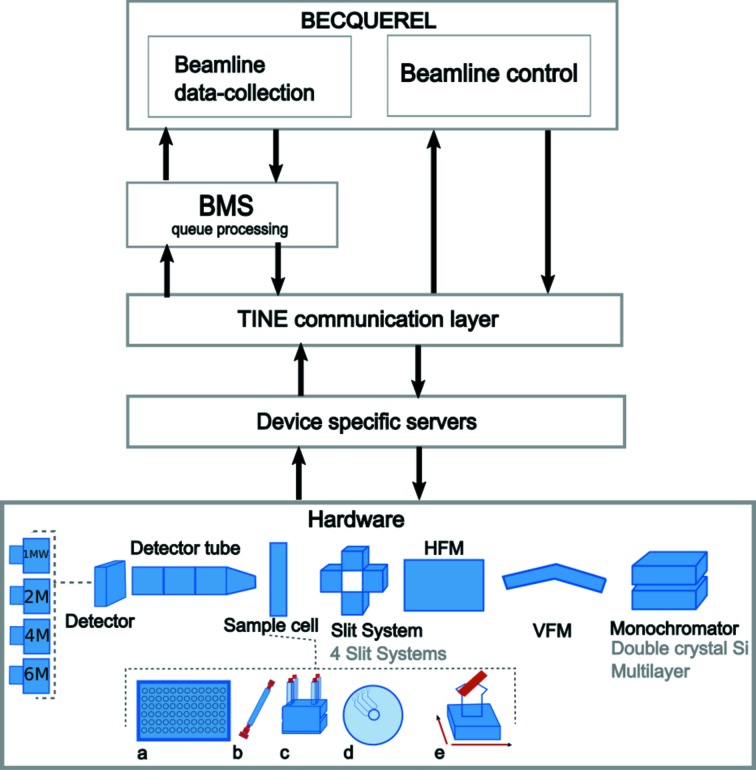
Schematic overview of the organization of the communication in *BECQUEREL*. The main interface has two major tasks: (i) controlling all devices at the beamline in a flexible manner and (ii) setting-up and triggering the data collection. All tasks, data collections and beamline optics are initiated through *BECQUEREL*, but an intermediary agent, the BMS, executes the commands during data collection. The communication is further mediated by an abstract communication layer, TINE, followed by the device specific servers that communicate directly to the hardware. *BECQUEREL* has different profiles for allowing smooth measurement and control of the different sample environments available at P12 (*a*, robotic; *b*, SEC-SAXS; *c*, stop-flow; *d*, microfluidic spinning disk; *e*, in-air samples). Abstraction in the representation of the hardware makes it easy to establish communication following a switch between, for example, the four detectors or the two monochromators available. HFM and VFM refer to horizontal and vertical focusing mirror, respectively.

*BECQUEREL* has been developed in C++ using the Qt libraries (https://www.qt.io) as its graphics toolkit. The communication to the low-level control occurs *via* the threefold integrated networking environment (TINE) servers at DESY, Hamburg (Bartkiewicz & Duval, 2007 ▸). *BECQUEREL* is only loosely coupled to TINE and can easily be substituted by another control system if needed (Fig. 1 ▸). In addition, a Python/C interface provides a user-accessible application programming interface (API) for convenient access to all devices and their commands through simple scripting (see §2.5). Below, we provide an overview of the general design concepts and functionalities of *BECQUEREL*.

### 2.1. General design   

*BECQUEREL* has, in accordance with good software development principles, separated the control code from the graphical representation. The GUI aims to hide most of the communication with the beamline components while maximizing the functionality. The design of the software is modelled on the fact that a beamline is essentially a linear assembly of hardware where downstream elements are dependent, to different extents, on the upstream components. All hardware devices at the beamline are represented as modular objects exporting properties, *e.g.* monitor and/or status readouts, commands and possibly sub-hardware(s). Here, sub-hardware(s) are either logically or physically dependent on the parent hardware. The implementation also encapsulates such interdependency; the successful initiation and execution of any command is contingent on the hardware executing the command, and may also be dependent on any number of separate devices. In *BECQUEREL*, these dependencies are part of the command itself by means of pre- and post-conditions, thus making it possible to execute a sequence of commands completely autonomously and in a timely fashion without requiring the user to explicitly specify the dependencies before every use.

### 2.2. Autonomous and nested command execution   

The actual command execution is handled by an auxiliary system, the beamline meta server (BMS), described in detail elsewhere (Franke *et al.*, 2012 ▸) (Fig. 1 ▸). Here, we outline a recent development, namely nested command queues and their applications. For example, advanced tasks, such as slit adjustment, are essentially the repetition of two commands: motor adjustment and monitor readout. Based on this, it is possible to hierarchically nest commands, thereby increasing the sophistication of the task. For instance, full slit positioning can be viewed as the sum of four blade positionings, which in turn is the sum of motor movements and monitor readings. In this fashion, one can increase the degree of command nesting and therefore the complexity of the task until, potentially, reaching full autonomous beamline alignment. Therefore, beamline optimization tasks such as slit adjustments as well as data collections of samples are nested. The nested command structure minimizes code redundancy and advances from the linearity of simple command execution, allowing for a reliable execution of complex tasks.

To successfully execute a nested command, *i.e.* full slit alignment, commands that analyse the results of the individual scans (*i.e.* fits) must be included and work robustly. In §3.4 we describe our strategy for the fitting procedure and how to auto-generate scans until the data can be reliably fitted. Finally, the ability to easily scale the complexity of the task could aid users with custom scans which need to modify their own workflow (§2.5); to this end we have added an example script (see the supporting information).

### 2.3. Continuous logging of status and monitor readings   

**Figure 2:**
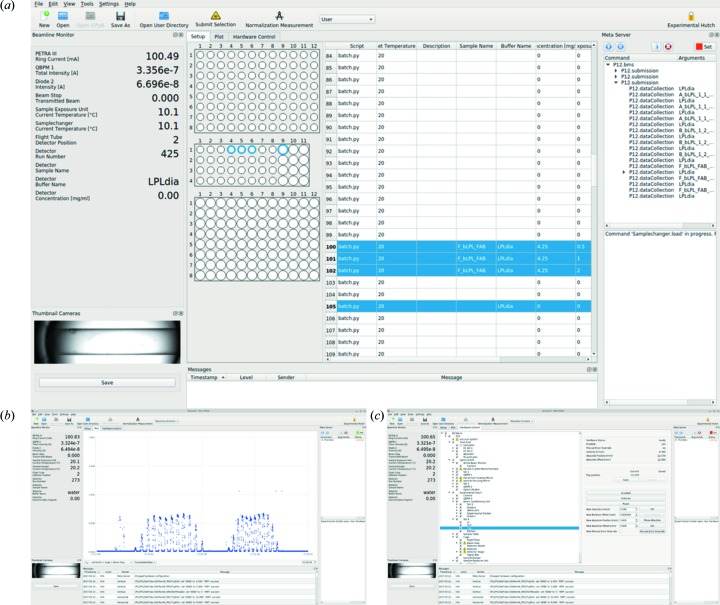
Screen-shot of the *BECQUEREL* GUI. (*a*) Left-most widgets give an overview of monitor readings specific to the profile, and a view of the sample holder (here, robotic sample-changer mode with a capillary view). The central widget accepts sample specifications of the user and displays the well structure of the sample holder. The upper right corner widget contains the status of the BMS and the current command queue. The queue can be paused, cleared and aborted (row of buttons). The Recommender widget (lower right corner) suggests commands and appropriate actions to take to allow for a data collection. Convenient shortcuts in the form of buttons are available in the top row, for submitting measurements and normalization measurements, breaking interlocks and accessing the data-collection directory. (*b*) Plot-tab, here showing transmitted beam over time during a robotic sample changer data collection. (*c*) Hardware tree-tab, with the hardware widget of a guard slit motor shown.

The properties of all hardware elements, *i.e.* their status and monitor readings, are continuously refreshed every 200 ms by the TINE sub-system and stored in memory for up to two hours since the given instance of *BECQUEREL* was started. This extensive logging provides three main functionalities that are useful for support staff as well as users. Firstly, the overall status of all the hardware and their properties can be manually exported at any time into a comprehensive .xml file. This functionality provides the beamline scientist with a safeguard in case previous positions of motors need to be recovered, and for users to recover information for comprehensive reporting and deposition of data (Trewhella *et al.*, 2017 ▸). In addition, the hardware status can be manually exported at any time through the ‘Export Beamline State’ in the ‘Tool’ item on the menu. Secondly, any previous value or reading can be retrieved for plotting (see §3.2, Fig. 2 ▸), making it easy to troubleshoot and to monitor change. Thirdly, continuously recording values also means that motor movements, such as those during a scan, are also logged. Hence, the scan windows (see §3.4) take advantage of this and simply retrieve the motor positions and monitor readings at the noted time-points. A clear advantage of such a structure is that, since all values are stored, it is easy to re-plot the scan values with another monitor reading without having to explicitly rescan.

### 2.4. Hardware control and access   

Apart from queued command execution, it is also possible to directly control the hardware through their hardware widgets. The hardware widgets are semi auto-generated for each hardware based on which commands are available and which properties can be configured. The semi auto-generated nature is due to the fact that not all properties are shown directly in the widget but may be converted on-the-fly into a human readable form. Thus some values have to be omitted on a case-by-case basis, otherwise a fully automated generation would be feasible. Like with queued command execution, the interdependency of each interactive action is checked to avoid conflicting actions in interactive hardware control. For example, it is not possible to manually flush a sample from the capillary while data collection is ongoing. It is, however, possible to override any dependency, *e.g.* during maintenance day, when the beamline is used for test purposes. The hardware widgets can be accessed through the hardware control tab (see §3.3).

Since the functionality of *BECQUEREL* includes both user data-collection and beamline alignment by the support staff, access modes have been implemented to delineate these two tasks. Three access modes are available: Observer, User and Beamline Scientist. In Observer mode, no actions can be executed, but the progress of the data collection can be passively viewed, for instance by a remote collaborator. User mode allows all actions directly related to a data collection, such as breaking interlocks and sample-environment adjustments. Scans and direct hardware control of upstream devices are only available through the password-protected Beamline Scientist mode.

### 2.5. Flexible automation through Python/C API scripting   

Once a device has been incorporated into the hardware library of *BECQUEREL*, an automatically generated generic Python/C API binding makes it possible to execute commands through Python scripting, in an interactive session, or as a script sent to the BMS for later execution. Indeed, all data-collections at P12 are executed through the Python scripts (Fig. 1 ▸). Standard data-collection methods such as robotic high-throughput or SEC-SAXS have custom scripts which are ready-made, and rarely, if ever, modified by regular users. Power users or support staff can modify these scripts to customize any data collection, or indeed any routine at the beamline. Most common uses of these scripts include scanning samples or any beam-conditioning element through the beam while triggering data collections. The ability to script any command of any device also makes it possible for support staff to write custom routines where many different devices are involved, such as optimizing the monochromator motor settings together with the undulator gap for adjusting the energy.

### 2.6. Recommender system to guide the troubleshooting process   

The code also implements a ‘goal state’ for the devices used in data collection. This is the condition for the device which will allow data to be collected, and allows the code to intelligently interpret error states. This feature, combined with the awareness of all device states and their values, makes it possible to identify which device (if any) has a problem and how to solve it. Such a procedure has been embodied in a troubleshooting routine called the Recommender. The Recommender suggests commands to be executed to prepare the beamline for measurements, or for when the data-collection unexpectedly pauses. The recommendations range from specific tasks such as restarting the detector to more general advice as to wait for the storage ring current to recover (Fig. 2 ▸).

## 3. *BECQUEREL* GUI   

Each experimental set-up comprises its own set of devices as well as its own sample-loading dynamics. With as many as five experimental set-ups in routine use at the P12 BioSAXS beamline, in addition to customized set-ups, easy switching between these is of utmost importance. *BECQUEREL* represents each set-up as a ‘profile’, which adapts the interface and the active devices accordingly (see §3.1). The active devices will in turn affect the scriptable properties (§2.5): for instance, changing the energy or sample flow-rate through a size-exclusion column can only be properly executed in the ASAXS or SEC-SAXS profile, respectively. The user is prompted to choose the ‘profile’ upon program initiation, and switching between profiles necessitates a new instance of *BECQUEREL*. Multiple instances of *BECQUEREL* can be open and active at the same time; however, only one profile can be registered with the BMS. For instance, it is possible to have two GUIs with SEC-SAXS and Batch profile open at the same time; however, only one of these instances will have its devices registered with the BMS, and therefore be operational. To register a profile with the BMS, it is necessary to simply click the button ‘Set’ (Fig. 2 ▸), where a green set button indicates an active profile. Finally, creating a new profile as well as adding new hardware is straightforward due to the modularity of *BECQUEREL*, in turn making it easy to accommodate custom sample-environments supplied by the user in advance of their beam time.

### 3.1. Overview   

*BECQUEREL* has a convenient interface to allow first-time users as well as support staff to navigate their respective tasks (Fig. 2 ▸). The interface devotes most of its screen estate to the central widget containing three tabs (see following sections), where the first and default tab represents the well arrangement of the sample environment. In this tab, the conditions of the experiment are specified; this is also where the most input from the user is expected (see §4). The central widget is encompassed by four smaller widgets, each encapsulating a particular functionality of the beamline. The beamline monitor displays the main monitor readings along the beam path and the relevant values for the experimental set-up in use, such as flow rate for SEC-SAXS or sample cell temperature for the robotic sample changer. Camera widgets show the sample environment, which in most cases is the on-axis sample-exposure unit camera with a view of the capillary. The sample-loader well arrangement, beamline monitors and camera widgets are all adjusted depending on the profile of *BECQUEREL.* The nested queue progression is encapsulated in a widget, with functionality to pause and start the queue. On the same widget, the button set hardware registers all devices of the profile with the command execution server, BMS. Finally, below the queue progression is the fourth and final widget containing the suggestions of the Recommender, as described in §2.6.

### 3.2. Plot tab   

The continuous logging of all values and readings in *BECQUEREL* (§2.3) can be visualized through the plot tab in the central widget (Fig. 2*b* ▸), which plots any monitor or device status over time. All values are stored for up to two hours after collection with the additional possibility to adjust the *x*-axis to home in on a certain time range. As a result, effects of adjustments in motors or optical elements can easily be visualized without the need of actively ‘collecting the data’. For users, plots of RI, UV or transmitted beam values of their samples over time can provide a convenient way to monitor their samples during long and continuous data-collection runs.

### 3.3. Hardware control tab   

At the P12 beamline the flexibility needed to accommodate many set-ups demands a convenient way for the beamline scientist to control the hardware directly through a clean and minimalistic interface. This is provided through the hardware control tab, which visualizes all the devices along the beamline, from the storage ring to the detector, and allows the direct control of the all the devices where (*a*) hardware commands are implemented and (*b*) the current user access level authorizes command execution (Fig. 2*c* ▸). Furthermore, a check-box next to each device determines whether it should be considered in the command execution. For example, when collecting data where the temperature of the sample-exposure unit is of no consequence, simply unchecking this device and re-registering the devices (through the set hardware button, Fig. 2 ▸) will permit unhindered command execution, even if the temperature is fluctuating. This feature also makes it possible to retain most of the automation of the data collection for custom set-ups, for which there is no dedicated ‘profile’ and data-collection script. In these cases, the sample environment will simply be unchecked, allowing the commands for the detector and the remaining devices to be triggered in a timely fashion.

### 3.4. Scan windows   

**Figure 3:**
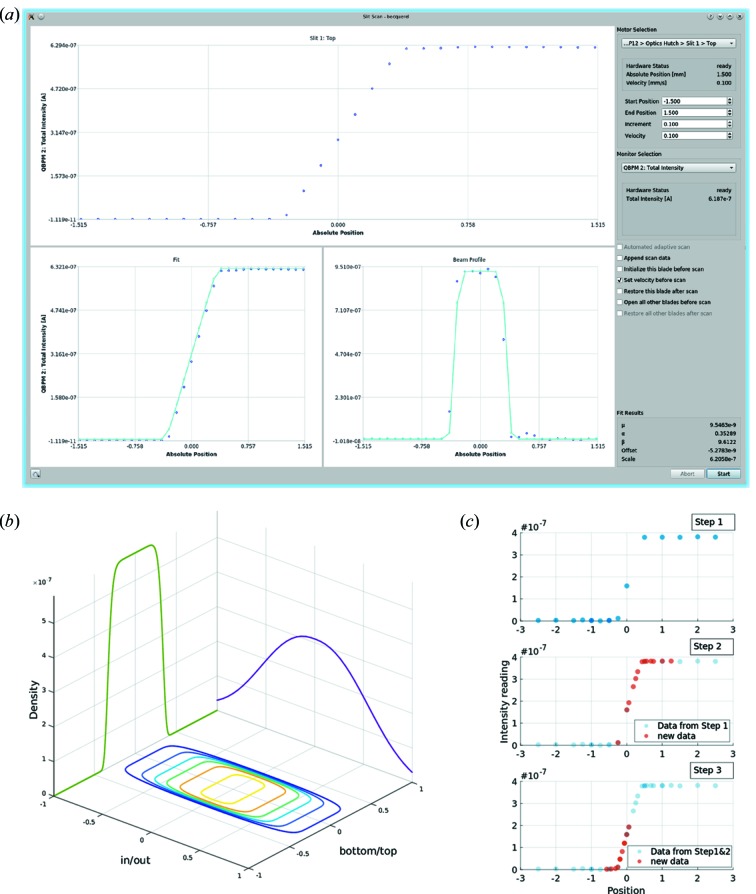
Overview of the slit scan functionality in *BECQUEREL* using the first slit after the monochromator. (*a*) Results of a slit scan with GGCDF and GGPDF fits to the experimental data and numerical derivatives. Re-scans can be made, and data appended to supplement the scan range. (*b*) Result of a fully automated positioning of a slit system. Data represented as beam profiles described by GGPDF are plotted in MATLAB. (*c*) Data from an adaptive scan (three steps) of one slit blade.

Common tasks for support staff include adjustments of optical elements such as slits and monochromators through various types of scans, accessible only in Beamline Scientist mode (*cf*. §2.4). Each scan-dialog window contains, apart from the set-up page (Fig. 3*a* ▸), a command queue-window, which displays the executed, currently running and scheduled commands in the scan. A description of the types of scans available is presented below.

#### 3.4.1. Linear and raster scans   

**Figure 4:**
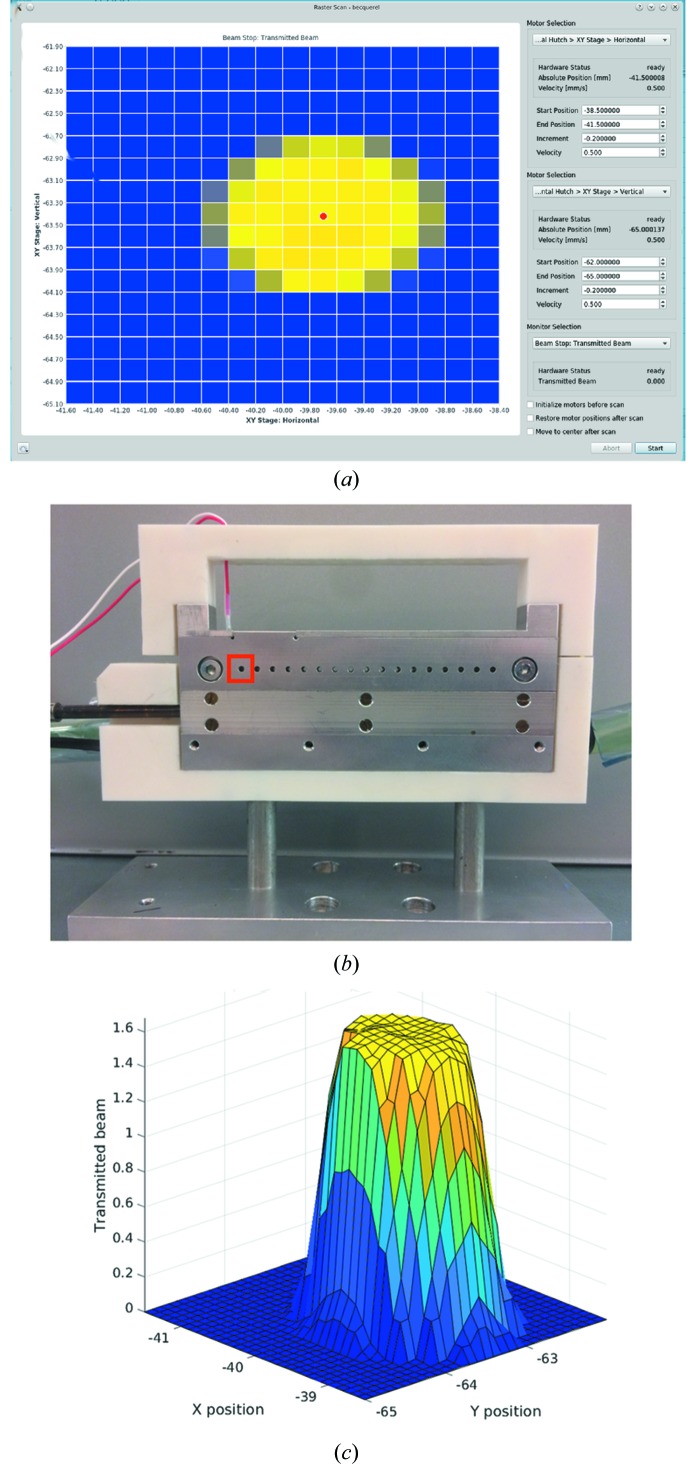
Example of a 2D scan, here of a custom *XY* stage containing 17 wells. (*a*) The 2D scan was performed to locate the first well [panel (*b*), red box], quickly position the sample stage and to extrapolate the position of the remaining wells. (*b*) Photograph of the 17 well sample-holder; the red box indicates the 2D scan area. (*c*) Corresponding data interpolated in MATLAB®.

Scans can either be along one- (1D) or two axes (2D) simultaneously, and are generalized such that any motor/s can be scanned against any monitor reading available at the beamline. Whenever a new motor or monitor is added to the beamline, it automatically becomes available in the list of optional devices for scanning. The monitor reading at the scan positions is directly plotted in real time (Fig. 3*a* ▸). In the case of raster scans of two axes, these are displayed as a heat map (Fig. 4*a* ▸). Furthermore, the data collected during any scan can be exported easily as .csv files for further data handling, for instance in MATLAB (Figs. 3*b*, 3*c* ▸ and 4*c* ▸). Finally, for the purpose of troubleshooting, it is also possible to initialize the relevant motors prior to the scan, or to restore the motor position after the scan.

The generality of these scans constitutes a powerful tool for quickly positioning a sample in a beam with an *XY*-stage (Figs. 4*a* and 4*b* ▸) or for robust large beam movements, for instance by 2D mirror pitches. Furthermore, the nested structure of the command execution invites the prospect of combining consecutive scans themselves in a nested fashion, allowing for shorter stretches of automatic alignment. There is no limit on the number of tasks that can be nested, and in theory tasks can be nested until reaching full alignment.

#### 3.4.2. Slit scans and fits   

Slit scans are essentially 1D scans, with the additional feature that the output of such scans is expected to be sigmoidal, where the sigmoidal fit can in turn be differentiated to find the beam center as well as an optimal position of the slit. This feature, together with the fact that the P12 beamline has a total of four slit systems, inspired the implementation of a customized slit scan dialog.

The slit scan dialog has all the functionality associated with general scans (§3.4.1), but with the addition that the output of each scan is fitted with a generalized Gaussian cumulative density function (GGCDF) (Nadarajah, 2005 ▸). The GGCDF consists of a family of exponential distributions, which includes the Laplacian, Gaussian and Uniform distributions. This feature makes GGCDFs well suited to accommodate different beam shapes, such as a top-hat or Gaussian, without making explicit assumptions about the shape. The parameters of the CDF (μ, α, β corresponding to the mean, scale and shape of the CDF) are obtained from the fit, and used to generate the related probability distribution function (PDF). This bell-shaped curve is analogous to the differentiated sigmoidal curve that is usually employed to find the optimal position of the slit-blade (Fig. 3*a* ▸). To our knowledge, this is the first reported use of GGCDFs for fitting slit-scan data, and we included MATLAB® (http://www.mathworks.com) scripts and example data for the interested user (supporting information).

Finally, the information gained by fitting a GGCDF can be used to guide further scans of the same system in a procedure called adaptive scanning. In short, the adaptive scan estimates the beam position from a rough initial scan and then, after fitting with a GGCDF, determines which regions have higher information content. Based on this, the scan procedure auto-generates more commands for rescanning these areas (Fig. 3*c* ▸) in a nested fashion. Adaptive scanning is therefore the unification of nested command structure and post-scan analysis, and provides a robust yet efficient way to perform full slit positioning (Fig. 3*b* ▸) completely autonomously.

## 4. Workflow of data collection with *BECQUEREL*   

To illustrate the autonomous data-collection, a general overview of the workflow is given below. It is intended to describe the full details of the data-acquisition process, and includes set-up procedures usually performed by the support staff. The actual required input of the user is limited, and outlined in §4.2.

### 4.1. Configuration and calibration measurements   

Prior to SAXS data collection, standard samples are measured, the forward scattering *I*(0) of which is used to determine the molecular weight (MW) of the actual samples. To obtain the scattering on an absolute scale (Jeffries *et al.*, 2016 ▸), water and its background, the empty capillary, can be measured. This procedure has been automatized at the P12 beamline for measurements in robotic sample-changer mode. Here, the user simply clicks the ‘Normalization Measurement’ button (Fig. 2 ▸) and the water and empty capillary is automatically measured. Water is extracted from the water tank of the robotic sample-changer, and while it is loaded an empty capillary shot is taken.

Under the ‘Settings’ item in the menu, the automatic data-reduction and analysis pipeline *SASFLOW* (Franke *et al.*, 2012 ▸) can be configured and started. In particular, the files for absolute calibration as well as the *I*(0) and MW of the standard protein are defined, such that *SASFLOW* may scale all subsequent data and determine the MW of the samples.

Note that there are additional calibration measurements that are performed during a change in detector distance or sample environment, such as beam-center determination and angular axis calibration using the scattering from a silver behenate powder. However, these calibration procedures are not performed exclusively through *BECQUEREL*, and an in-depth description of the methodology is available elsewhere (Huang *et al.*, 1993 ▸; Konarev *et al.*, 2003 ▸).

### 4.2. Measurement set-up   

The many measurement strategies and sample environments available at P12 demands an equally flexible yet intuitive measurement set-up procedure in *BECQUEREL*. Here, the user specifies the sample and buffer name in addition to more specific conditions such as exposure time, number of images, energy or cell-temperature for each sample source, or well. The settings that are available to customize for each well are largely dependent on the profile of *BECQUEREL* (§3.1). As each well can have its own set of parameters, it is easy to queue measurements of differing specifications such as energy or cell-temperature, depending on the profile being used. While the well specifications depend on the currently active profile, it is in principle always possible to queue measurements and/or movements of any device through the scripting capabilities (see §2.5). As such, it is very straightforward to automate procedures on-the fly. To measure one or more wells, it is sufficient to select the relevant wells and press the ‘Submit Selection’ button above the well plate (Fig. 2*a* ▸, highlighted). Before the commands are sent to the processing queue, the user-defined values are validated. Any invalid parameters that are identified are communicated instantly *via* a pop-up box and no measurements can be submitted until all values are valid.

In robotic sample-changer mode, buffer and sample measurements are automatically matched based on matching buffer-name, and the measurement sequence measures the buffer twice (Fig. 2*a* ▸). Finally, it is also possible to prepare experiment definitions beforehand through the information management system ISPyB (De Maria Antolinos *et al.*, 2015 ▸), and to import the prepared data into *BECQUEREL* when on-site. This becomes especially convenient for large batch set-ups and mail-in operation.

### 4.3. During data-collection   

Once the measurement has been submitted, the command script (§2.5) is run and the listed commands are queued for execution, thereby also concluding the need for any user input. The command queue (Fig. 2*a* ▸) gives an overview of all the finished, running and scheduled commands. The left-hand monitor updates with the details of the sample that is being measured. If there is an error, for instance beam-loss or shortage of available consumables, the system would pause and the Recommender (bottom right widget, Fig. 2*a* ▸) would suggest the most appropriate action(s) on how to commence the data collection. Multiple recommendations can be given, such that the user is walked through common hardware problems to make the beamline operational again.

During sample loading in robotic sample changer mode, a 1 s exposure of the empty capillary shot is collected just prior to the sample appearing in the capillary; radially averaged and superimposed empty capillaries profiles allow for monitoring the gradual build-up on the capillary. This procedure becomes especially pertinent when collecting radiation-sensitive and large proteins that can severely tax the quality of the capillary. Capillary build-up can be remedied by a hot clean, whereby the temperature of the capillary is increased to ∼50°C and detergent [we utilize 2% Hellmanex III, 10% ethanol and 88% distilled water (Round *et al.*, 2015 ▸)] is loaded and kept for approximately 30–40 minutes.

## 5. Conclusion   

We have given an overview of the latest developments of the beamline control and data-acquisition software for biological SAXS, *BECQUEREL*, in operation at the P12 beamline since 2011. *BECQUEREL* is written in C++ and a Python interface for easy access and scripting of hardware commands, a combination that utilizes the cross-platform compatibility and stability with the user-friendliness from each language, respectively. Through an overview of its functionalities we stress the convenience of uniting beamline control and data-acquisition in one interface, especially in the context of streamlining the switch between experimental set-ups. The modularity of *BECQUEREL* makes it possible to not only distance it from the low-level control but also to separate the data-collection and command execution from the beamline control part. Despite the extended functionalities of *BECQUEREL*, the measurement set-up and operation procedure remains highly intuitive as to allow novice users to begin the data collection within a few minutes of having received an introduction by the beamline support staff. It is also possible to access *BECQUEREL* from remote computers making it straightforward to conduct remote measurements at P12. In the standard remote access mode, the users simply send 96-well plates with the samples, which are placed into the robotic sample changer by the beamline staff and the measurements are conducted from the user’s computers at their location.

The version of *BECQUEREL* described here has been in operation at the P12 beamline for approximately one year, during which it has seen over 100 user groups and a thorough use of its beamline control and scan functionalities, especially during three beam commissionings. In total, *BECQUEREL* has been operational at the P12 beamline for six years, and therefore its basic functionalities have seen a thorough testing and continuous improvement. In the past year, experiments that utilized the extended functionalities of *BECQUEREL* included high-throughput automated sample delivery measurements (Pedersen *et al.*, 2017 ▸), SEC-SAXS measurements with additional biophysical characterization (Pflüger *et al.*, 2018 ▸), in-air measurements (Haenelt *et al.*, 2018 ▸), high-flux time-resolved measurements and also anomalous SAXS studies..

## Supplementary Material

MATLAB script to fit a GGCDF to, for instance, a slit scan.. DOI: 10.1107/S1600577518005398/ay5515sup1.txt

MATLAB script to fit a GGPDF (probability distribution function) and returning the FWHM of the fit. DOI: 10.1107/S1600577518005398/ay5515sup2.txt

MATLAB script for fitting an inverse GGCDF . DOI: 10.1107/S1600577518005398/ay5515sup3.txt

Example Python script that automates a part of an alignment procedure. DOI: 10.1107/S1600577518005398/ay5515sup4.txt
